# Hysteroscopic sterilization in immunocompromised patients who have intrauterine devices in place: two case reports

**DOI:** 10.1186/s13256-015-0729-y

**Published:** 2015-10-28

**Authors:** Camille Ladanyi, Carlie Field, Kristina Tocce

**Affiliations:** 1 Department of Obstetrics and Gynecology, Maine Medical Center, 22 Bramhall Street, Portland, ME 04102 USA; 2 Department of Obstetrics and Gynecology, University of Washington School of Medicine, 1959 NE Pacific Street, Seattle, WA 98195 USA; 3 Department of Obstetrics and Gynecology, University of Colorado Anschutz Medical Campus, 12631 E. 17th Avenue, Room 4006, Aurora, CO 80045 USA

**Keywords:** Essure, Hysteroscopic sterilization, Immunosuppression

## Abstract

**Introduction:**

The micro-inserts used in the hysteroscopic sterilization procedure elicit a benign occlusive tissue response leading to permanent tubal occlusion. Little is known about whether immunosuppressed patients mount the immunological response necessary to ensure tubal occlusion. Theoretical concern for non-occlusion has limited the use of hysteroscopic sterilization in patients on immunosuppressive therapies.

In all patient populations, if an intrauterine device is in place, it is usually removed at the time of hysteroscopic sterilization. Little is known about maintaining intrauterine devices during the 3-month period to tubal occlusion.

**Case presentation:**

Our patient in case 1 was a 35-year-old Hispanic woman, gravida 2, para 2002, with a history of a living donor kidney transplant. Our patient in case 2 was a 32-year-old Hispanic woman, gravida 3, para 2103, diagnosed with undifferentiated autoimmune disease. Both patients underwent hysteroscopic sterilization. In both cases, a levonorgestrel intrauterine device was in place for contraception. At the time of micro-insert placement, our patients were both on daily immunosuppressive medications, including long-term glucocorticoids. Three months after the hysteroscopic procedure, both patients had successful tubal occlusion, demonstrated by a hysterosalpingogram.

**Conclusion:**

Hysteroscopic sterilization in an outpatient setting is a reasonable option for sterilization in immunocompromised patients on immunosuppressive therapies. Intrauterine devices can be maintained during the procedure and during the 3-month period to tubal occlusion.

## Introduction

The Essure® permanent birth control system (Bayer HealthCare Pharmaceuticals Inc., Whippany, NJ, USA.) was first approved by the US Food and Drug Administration in 2002 and has since been used in approximately 450,000 sterilization procedures worldwide [1]. The Essure® system is an outpatient hysteroscopic sterilization procedure that provides permanent contraception through transcervical hysteroscopic placement of micro-inserts into the proximal portion of each fallopian tube. The system has a 99.74 % effectiveness rate with a 0.26 % failure rate due to device expulsion, uterine perforation at the time of device placement, improper placement of the device, lack of appropriate follow-up, and luteal phase pregnancies prior to device placement [1]. Each Essure® micro-insert is 40 mm in length and 0.8 mm in diameter and consists of a stainless steel inner coil, a nickel titanium elastic outer coil, and polyethylene terephthalate fibers. When released from the delivery system, the outer coil expands to 1.5–2.0 mm to anchor the insert into the fallopian tube [2]. Over time, the polyethylene terephthalate fibers within the micro-insert trigger a benign chronic inflammatory and fibrotic response which results in permanent tubal occlusion [3]. Additional birth control is needed until tubal occlusion is confirmed at 3 months by a hysterosalpingogram (HSG) [1].

Histologically, the polyethylene terephthalate fibers produce an immediate local inflammatory response with macrophages, fibroblasts, foreign body giant cells, and plasma cells [3]. A foreign body inflammatory reaction is also elicited by the polyethylene terephthalate fiber, which peaks after the device has been in place for 2–3 weeks. After the device is in place for 8–30 weeks, chronic inflammation and fibrosis predominates the tissue response. Ultimately, strong fibrotic and inflammatory responses between the inner and outer coil of the micro-insert lead to ingrowth of loose and dense fibrous tissues, producing tubal occlusion and permanent sterilization [3].

Currently, the instructions for use included in each Essure® kit contain the following warning: “Patients undergoing immunosuppressive therapy are discouraged from undergoing the Essure® procedure because the immunosuppressant may lead to decreased tissue in-growth” [4]. These theoretical concerns regarding efficacy of hysteroscopic sterilization cause this method of sterilization to be underutilized in a patient population that could greatly benefit from a minimally invasive option. Current literature includes only one case report that documents a successful hysteroscopic sterilization in a 38-year-old kidney transplant recipient who was on mycophenolate (CellCept) 250 mg twice a day and sirolimus 2 mg daily [5]. To the best of our knowledge, there is no literature on immunocompromised patients who had hysteroscopic sterilization and maintained their previously placed levonorgestrel (LNG) intrauterine device (IUD) during the time to tubal occlusion.

In a retrospective case review of 174 patients receiving Essure® sterilization at the University of Colorado Hospital between February 2008 and August 2013, two patients were on immunosuppressive therapies at the time of the procedure. In this report, we present these two patients who had HSG-confirmed successful hysteroscopic sterilization with previously placed LNG IUDs in place.

## Case presentations

### Case 1

Our patient was a 35-year-old Hispanic woman, gravida 2, para 2002, with a history of a living donor kidney transplant at age 23 secondary to hypertensive nephrosclerosis. Her maintenance immunosuppressive therapy included mycophenolate mofetil (CellCept) 1000 mg twice a day, prednisone 5 mg daily, and tacrolimus 3 mg twice a day. After her second pregnancy was complicated by A2 gestational diabetes, she wanted permanent sterilization. However, her gynecologist felt that her immunosuppression was a contraindication to micro-insert placement and she had a LNG IUD placed instead. After 5 years of LNG IUD use, she continued to want hysteroscopic sterilization. She had normal kidney function and was stable on her medication regimen. After consultation and a thorough review of the risks of the procedure at our University-based Family Planning Clinic, our patient was scheduled for hysteroscopic sterilization in the outpatient setting with oral sedation (hydrocodone 10 mg and lorazepam 1 mg). Ketorolac was not administered owing to her history of renal transplant.

The Essure® procedure was uncomplicated; two coils were visualized in her uterine cavity following left-sided placement and four coils were visualized on her right side. The procedure was done with the LNG IUD in place. Prophylactic antibiotics were not administered. Three months later, HSG showed bilateral occlusion of her fallopian tubes and the LNG IUD was subsequently removed.

### Case 2

Our patient was a 32-year-old Hispanic woman, gravida 3, para 2103, with a history of undifferentiated autoimmune disease and polymyositis, diagnosed 17 months prior to the hysteroscopic sterilization procedure. She was on mycophenolate mofetil (CellCept) 1500 mg twice a day and methylprednisolone 2 mg twice a day. Our patient was originally started on methylprednisolone 16 mg twice a day 1 year prior to her hysteroscopic sterilization procedure, which was subsequently tapered to 2 mg daily by 1 month after the sterilization procedure. Her autoimmune disease and polymyositis symptoms were well controlled with this medication regimen. Results from laboratory tests were also markedly improved, including estimated sedimentation rate and creatine kinase level, at the time of procedure. Our patient had a LNG IUD in place for 5 years with no adverse effects, but wanted permanent sterility. After consultation at our University-based Family Planning Clinic, our patient was scheduled for hysteroscopic sterilization in the outpatient setting with oral sedation (hydrocodone 10 mg and lorazepam 1 mg) and 60 mg of intramuscular ketorolac.

Micro-insert placement was uncomplicated; three coils were visualized in her uterine cavity following both left-side and right-side placement. The procedure was done while leaving the LNG IUD in place. Prophylactic antibiotics were not administered. Three months later, HSG showed bilateral occlusion of her fallopian tubes and the LNG IUD was subsequently removed.

## Conclusions

These cases demonstrate successful tubal occlusion with the hysteroscopic sterilization system in two patients taking immunosuppressive medications (B and T lymphocyte inhibitors and chronic steroids). Each patient used a LNG IUD prior to the procedure, and the IUDs were left in place during the time to tubal occlusion. There were no adverse events related to maintaining the IUDs. Bilateral tubal occlusion was confirmed in both patients at 3 months with HSG.

Bilateral tubal occlusion with the Essure® system relies on the patient’s ability to mount a foreign body reaction to the polyethylene terephthalate fibers in the coil [3]. Owing to theoretical concerns that immunosuppressants will interfere with this reaction, hysteroscopic sterilization in immunosuppressed women is discouraged by the manufacturer. However, the foreign body reaction consists primarily of macrophages, fibroblasts, and foreign body giant cells [3]. Immunosuppressive medications are thought to have minimal effect on macrophage activity and the other cell types involved are not typical targets of most immunosuppressive therapies. Most immunosuppressive therapies prevent the proliferation and activation of B and T lymphocytes of the adaptive immune system [6]. Therefore, these medications should not interfere with the foreign body response to the micro-inserts or the chronic inflammation and fibrosis that leads to tubal occlusion. In the two cases presented here, both patients were on medications that are thought to inhibit the proliferation of B and T lymphocytes. In the aforementioned case report from 2012, successful tubal occlusion was demonstrated in a patient on mycophenolate mofetil (CellCept) 250 mg twice a day and sirolimus 2 mg daily, both B and T cell suppressants [5]. Unlike our patients, this patient was not taking glucocorticoids.

Systemic glucocorticoids affect the immune system by inhibiting the transcription of various pro-inflammatory cytokines and promoting the transcription of anti-inflammatory cytokines. Because foreign body cells are responsive to cellular signals and participate in the inflammatory response through production of cytokines, glucocorticoids could theoretically alter the foreign body reaction to the micro-inserts [7]. However, both patients presented in this report were on chronic glucocorticoids and they mounted the appropriate immune response to the micro-inserts. It is likely that with the multitude of different cytokines produced during a foreign body reaction, sufficient pro-inflammatory cytokines are still present and occlusion of the tubes is successful. In addition to the foreign body response, the micro-inserts also produce chronic inflammation and tissue in-growth with fibrosis, both of which contribute to permanent sterilization [3].

Organ transplant recipients and patients with autoimmune disorders are at increased risk of pregnancy-related complications, including Cesarean section, gestational diabetes, preeclampsia, preterm delivery, small for gestational age, intrauterine growth restriction, and intrauterine fetal death [6, 8, 9]. Hysteroscopic sterilization in the outpatient setting allows these patients with medical complications to obtain sterilization while avoiding additional surgery and general anesthesia. Hysteroscopic sterilization should be offered as an option for these patients. Medications that inhibit B and T cell proliferation should not affect efficacy. Steroid use can be presented as a *theoretical* risk for non-occlusion. However, in these two patients on glucocorticoids, adequate tissue in-growth occurred and bilateral tubal occlusion was seen. More research is needed to further elicit the effects of specific steroids and their dosages on the tissue response to micro-insert placement.

Continuing to use another form of contraception until bilateral tubal occlusion is documented by HSG is paramount in all patients who undergo hysteroscopic sterilization. These two cases not only demonstrate that hysteroscopic sterilization can be successful in patients on immunosuppressant therapies, but that IUDs can be maintained during and after the hysteroscopic sterilization procedure. As with many patients with medical complications, avoiding additional surgery and general anesthesia makes the hysteroscopic approach to sterilization attractive. The use of this sterilization technique, and maintenance of IUDs until tubal occlusion is documented, should be considered a viable option for patients on immunosuppressive therapies.

## Consent

Written informed consent was obtained from the patients for publication of this case report. A copy of the written consent is available for review by the Editor-in-Chief of this journal.

## Abbreviations

HSG: hysterosalpingogram
LNG: levonorgestrel
IUD: intrauterine device
